# Research on the Impact of Challenge-Hindrance Stress on Employees’ Innovation Performance: A Chain Mediation Model

**DOI:** 10.3389/fpsyg.2022.745259

**Published:** 2022-04-05

**Authors:** Wei Cai, Chao Xu, Shengxian Yu, Xiaoxiao Gong

**Affiliations:** ^1^Faculty of Education, Universiti Kebangsaan Malaysia, Kuala Lumpur, Malaysia; ^2^School of Finance, Hubei University of Economics, Wuhan, China; ^3^School of Business Administration, South China University of Technology, Guangzhou, China; ^4^School of Business Administration, Southwestern University of Finance and Economics, Chengdu, China

**Keywords:** challenge stress, hindrance stress, intrinsic motivation, knowledge acquisition, innovation performance

## Abstract

Based on the transaction theory of stress and the theory of resource conservation, which introduces knowledge acquisition and intrinsic motivation as mediating variables, a chain mediating model for the influence of challenge-hindrance stress on innovation performance is constructed. Data of 295 samples collected in three stages were used to testify hypothesis. The results confirmed a positive relationship between challenge stress and innovation performance, and a negative relationship between hindrance stress and innovation performance. Intrinsic motivation and knowledge acquisition play a parallel and chain mediating role in the relationship between challenge-hindrance stress and innovation performance. These findings contribute to a deeper understanding of how challenge -hindrance stress affects innovation performance and provide important practical guidance for improving innovation performance.

## Introduction

With the rapid development of artificial intelligence and digital economy, innovation has become key to the sustainable development of organizations (Zhou and George, 2003). As the backbone of innovation productivity, employees’ innovation performance is directly related to the development of organizations. Regarding the influencing factors of employees’ innovation performance, previous research has mainly focused on individual and organizational levels. From an individual perspective, such as the impacts of psychological capital and self-efficacy on employee innovation (Gong et al., 2012; Acar et al., 2019). At the organizational level, leadership style, organizational climate, and organizational incentive systems impact employees’ innovation performance (Hon et al., 2013; Massis et al., 2018). Certain achievements have been made regarding the influencing factors of innovation performance; however, the current situation of organization management and employment environment has undergone significant changes (Nikolova et al., 2019; Yun et al., 2019). The changing environment places higher demands on creative skills and psychological quality of employees, who will face more uncertainties and job stress in their work (Yu et al., 2021). Based on this, the present study focuses on how job stress affects innovation performance in new work situations and how the mechanisms of influence can be further explored.

At present, role ambiguity, workload, time conflict, and workplace exclusion are prevalent in organizations, all of which impose mental tension and stress on employees (Yu et al., 2021). In previous studies, job stress has been regarded as an important factor affecting employee innovation, but two shortcomings remain. First, there are contradictory conclusions in previous research. Specifically, the results can be divided into four types: Promoting effect (Ohly and Fritz, 2010), inhibiting effect (Hon and Lui, 2016), inverted U-shaped effect (Baer and Oldham, 2006), and irrelevant (Amabile and Gryskiewicz, 1989). Second, most studies only examined the direct relationship between job stress and innovation performance, while the mechanisms between the two have been rarely explored. Based on the above analysis, a fundamental theoretical problem becomes inevitable: How can job stress have a differential impact on innovation performance? In this context, the transactional theory of stress provides a powerful explanation for the relationship between job stress and innovation performance (Lazarus, 1991). According to the duality of stress, job stress can be divided into two types: challenge stress and hindrance stress (Cavanaugh et al., 2000). Previous studies have indicated that challenge stress and hindrance stress have positive and negative effects, respectively, on behavioral outcomes (Lepine et al., 2005; Webster et al., 2011). Whether the nature of stress is the reason for this differential effect of job stress on innovation performance requires further analysis.

The influence mechanism of job stress on innovation performance is still not effectively explained. In the present study, intrinsic motivation and knowledge acquisition are used as mediating variables. First, although scholars generally agree that intrinsic motivation is an important driving force of innovative behavior and creativity (Granta and Berry, 2011; Zong et al., 2016), intrinsic motivation has not been explored from the perspective of the nature of job stress. Intrinsic motivation is a positive psychological resource, which refers to the individual’s desire to work hard based on the interest in work (Deci and Ryan, 1985). Based on the theory of resource conservation (Hobfoll, 1989), the type of stress has a differential effect on intrinsic motivation. When dealing with challenging job requirements, employees are intrinsically motivated to complete tasks and thus invest more resources toward achieving performance goals. Hindrance job requirements can cause employees to experience frustration, fear, or even depression, all of which reduce the intrinsic motivation for resource investment and causes employees to adopt defensive behaviors (Rodell and Judge, 2009). In view of the above, this study suggests that challenge-hindrance stress will have a differentiated impact on intrinsic motivation, thereby affecting innovation performance.

Second, knowledge acquisition refers to the process in which employees acquire new knowledge from the outside (Husted and Michailova, 2002), which is an important factor affecting employee innovation. However, according to an analysis of the existing literature, few scholars have focused on the impact of job stress on knowledge acquisition. Based on the theory of resource conservation, hindrance stress can weaken employees’ confidence and expectations regarding task completion, which in turn can affect resource investment in knowledge acquisition and inhibit innovation performance levels (Cerne et al., 2017; Wang et al., 2019). Therefore, it stands to reason whether knowledge acquisition can act as a bridge between challenge-hindrance stress and innovation performance. This is worthy of further discussion. Finally, this study explores the chain mediating role of intrinsic motivation and knowledge acquisition in the relationship between challenge-hindrance stress and innovation performance. This study helps to clarify the mechanism of job stress on innovation performance, and provides insights for organizational managers to effectively cope with job stress and improve employees’ innovation performance.

**FIGURE 1:**
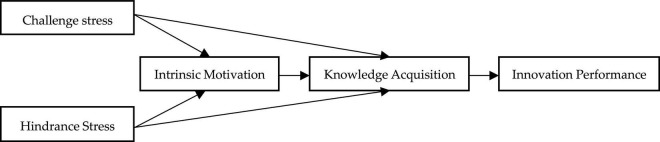
Theoretical model.

This study presents a number of theoretical contributions. First, previous studies mainly analyzed the outcome effect of job stress from a single perspective (e.g., Wallace et al., 2009; Montani et al., 2017), but it remains unclear whether job stress has a double-edged sword effect. From the perspective of the nature of job stress, it can be confirmed that challenge-hindrance stress has different impact effects on innovation performance. The findings of this study identify the reasons for this double-edged sword effect of job stress and offer a way to overcome the limitations of previous studies. Second, few studies have explored the impact mechanism of challenge-hindrance stress on innovation performance, especially from the perspective of motivation. This study confirms that intrinsic motivation plays a mediating role between challenge-hindrance stress and innovation performance. The findings expand the perspective of the mediating mechanism between challenge-hindrance stress and innovation performance. Third, previous studies mainly used knowledge acquisition as a dependent variable (Wang and Zhang, 2010; Martinez et al., 2012), while its mediating role has not been analyzed. This paper confirms that knowledge acquisition plays a mediating role in the relationship between challenge-hindrance stress and innovation performance. This enriches the research perspective of knowledge acquisition and mediating mechanisms in the relationship between challenge-hindrance stress and innovation performance. Finally, it is confirmed that intrinsic motivation and knowledge acquisition play a chain mediating role between challenge-hindrance stress and innovation performance. These findings enrich the understanding of the logical relationships among antecedent variables of innovation performance and provide a reference for variable selection in subsequent studies. According to the above analysis logic, the theoretical model of this study is shown in Figure 1.

## Literature Review and Hypotheses

### Challenge-Hindrance Stress and Innovation Performance

Based on the dual nature of stress, job stress can be classified as challenge stress and hindrance stress. Challenge stress refers to job stress that is regarded as surmountable and beneficial to performance and personal growth, and includes time stress, job overload, and job responsibilities (Cavanaugh et al., 2000). Hindrance stress refers to stress individuals find difficult to overcome and that prevents them from achieving job goals and career growth, including role ambiguity, organizational politics, and job insecurity (Cavanaugh et al., 2000). Challenge stress has been shown to have a significant positive impact on work behavior (Rodell and Judge, 2009; Sillai and Gamero, 2014). On the contrary, as a threatening negative job requirement, hindrance stress can disrupt an individual’s mental and emotional state, thus tends to negatively impact work behavior (LePine et al., 2004; Webster et al., 2010). In summary, the different natures of job stress types usually lead to differentiated results.

Innovation performance refers to novel and useful ideas, methods, procedures, or new products produced by employees (Janssen and Yperen, 2004). Based on the above challenge-hindrance stress effect logic, the impact of challenge-hindrance stress on innovation performance should also be different. First, according to the transactional theory of stress, employees assess external stress based on the interaction between their own abilities and the work situation, which in turn affects their psychological perceptions (Yun et al., 2019). The individual has the capacity to deal with challenge stress, and doing do helps to improve their own abilities and performance levels. Therefore, employees usually hold positive expectations for challenge stress and adopt problem-oriented coping strategies, thus mobilizing their individual subjective ability to promote the formation of innovative ideas. In contrast, hindrance stress can interfere with the achievement of employees’ task goals and cause employees to perceive insurmountability (Pearsall et al., 2009; Peng et al., 2019). Thus, under the influence of hindrance stress, employees tend to choose defensive measures and find it difficult to generate new ideas. Second, according to the theory of resource conservation (Hobfoll, 1989), individuals are willing to invest existing resources into high-return activities to increase their own resource stock. Therefore, employees tend to consume resources such as time, energy, and social relations to face challenge stress that can bring more benefits. Clearly, positive resource input is more likely to produce positive results, i.e., challenge stress can effectively enhance employees’ creativity (Espedido and Searle, 2020). On the contrary, hindrance stress causes employees to consume considerable emotional and emotional resources in their work (Yun et al., 2019). To avoid further loss of resources, employees try to adopt resource defenses, which exerts a certain inhibitory effect on innovation performance. Finally, related studies also proved that there are differences in the outcome effects of challenge-hindrance stress. Hon et al. (2013) found that challenge stress has a significantly positive impact on creativity, while hindrance stress has a significantly negative impact on creativity. Moreover, job stress can affect employees’ innovation motivation through emotion. While challenge stress can give employees a sense of meaning in their work and thus stimulate their innovation motivation, hindrance stress can negatively affect emotional states and inhibit innovation motivation (Montani et al., 2017). Based on the above analysis, the following hypotheses are proposed:

***Hypothesis 1a:*** *Challenge stress is positively related to innovation performance.*

***Hypothesis 1b:*** *Hindrance stress negatively related to innovation performance.*

### Mediating Effect of Intrinsic Motivation

To identify the influence mechanism of the relationship between challenge-hindrance stress and employees’ innovation performance, intrinsic motivation is introduced as a mediator. Intrinsic motivation has been considered as a key factor for predicting employees’ innovation (Ryan and Deci, 2000; Shalley et al., 2004). When employees are motivated by the organization, they are more willing to convert their motivation into work effort, especially for job tasks that require creativity, cognitive flexibility, and understanding (Hau et al., 2013). Moreover, employees with a high level of intrinsic motivation tend to be persistent in the face of difficulties, and more focused on seeking solutions to problems (Kehr, 2004; Rodell and Judge, 2009), thereby more likely to showing creativity. Intrinsic motivation can effectively stimulate the flexibility and sensitivity of individual thinking, and thus promote the formation of innovative ideas (Amabile and Conti, 1999; Min et al., 2015). Therefore, intrinsic motivation has a significant impact on innovation performance. Zhang and Bartol (2010) found that an individual’s intrinsic motivation is conducive to increased job competence and engagement, thus contributing to improved innovation performance. According to the above analysis, as a kind of work environment cognition, challenge-hindrance stress also likely affects innovation performance through intrinsic motivation.

Specifically, challenge-hindrance stress has a differentiated impact on employees’ intrinsic motivation, thus affecting innovation performance. The potential benefits of challenge stress can stimulate employees’ sense of self-efficacy and contribute to their career development, thus promoting their intrinsic motivation for innovation (Hau et al., 2013). Hindrance stress prevents the growth of employees making them experience insurmountable stress, thus inhibiting their motivation to innovate (Wallace et al., 2009). According to the transactional theory of stress, when employees cannot overcome hindrance events, they easily experience negative emotions and escape psychology, thus inhibiting their intrinsic motivation to work (Min et al., 2015). Drawing on theory of resource conservation, when faced with challenge events, employees show both positive attitude and intrinsic motivation, expecting to obtain benefits by completing job tasks (Hobfoll, 1989). Hindrance stress leads to psychological tension and negative perceptions among employees, thus creating an intrinsic motivation to protect vested resources, which in turn inhibits innovation performance (Dawson et al., 2016; Wood and Michaelides, 2016). For employees, challenge stress is beneficial as it increases flexible working space and initiative to explore problems (Montani et al., 2017), stimulates employees’ intrinsic motivation to overcome difficulties, and improves their innovation performance. In contrast, hindrance stress can lead to role ambiguity and strengthen the perception of being marginalized in the organization, which in turn reduces intrinsic motivation and subsequent innovation performance (Sacramento et al., 2013). In addition, long-term hindrance stress will also induce a lasting feeling of helplessness in employees, thus reducing their intrinsic motivation and lowering their performance (Widmer et al., 2012). In summary, this study puts forward the following hypotheses:

***Hypothesis 2a:*** *The positive association of challenge stress on innovation performance is mediated by intrinsic motivation.*

***Hypothesis 2b:*** *The negative association of hindrance stress on innovation performance is mediated by intrinsic motivation.*

### Mediating Role of Knowledge Acquisition

Knowledge acquisition is a process of the consumption and acquisition of resources, which reflects the acquisition of new knowledge or skills through interaction among different knowledge subjects (Akhavan and Hosseini, 2016). This study predicts that knowledge acquisition mediates the relationship between challenge-hindrance stress and innovation performance. First, challenge-hindrance stress impacts knowledge acquisition, as knowledge acquisition goals and strategies are affected by individual cognitive factors (Chuang et al., 2016). Overcoming challenge stress can yield lucrative rewards for individuals, including performance and job promotions. Thus, individuals will seek external knowledge and skills to solve stressful dilemmas with a more positive attitude (Cerne et al., 2014; Tayyaba et al., 2020). On the contrary, job insecurity caused by hindrance stress makes individuals more inclined to adopt conservative strategies to prevent resource depletion and thus leading them to not invest too much resources into knowledge acquisition (Woodfield and Husted, 2017; Tayyaba et al., 2020). In other words, hindrance stress has an inhibitory effect on employees’ knowledge acquisition (Hobday et al., 2012). Moreover, when challenged with stressful situations, it is often impossible for individuals to have all resources needed to complete job tasks and goals. To complete job tasks and goals, individuals must inevitably supplement external resources, which to some extent stimulates the motivation to acquire knowledge (Drees and Heugens, 2012). However, when faced with hindrance stress, individuals usually choose to give up their task goal and lack the motivation to acquire knowledge, thus inhibiting the resource output of knowledge acquisition (Drees and Heugens, 2012). Moreover, previous scholars have also pointed out that knowledge acquisition has a significant positive impact on both innovative behavior and creativity (Liu et al., 2017).

Second, knowledge acquisition positively impacts innovation performance. Studies have shown that knowledge acquisition has a significant positive impact on innovation performance at the organizational and group level (Liu et al., 2016). However, both organizational performance and team performance are the result of individual performance. In the knowledge and information era, it is difficult for employees to provide the high cost of knowledge innovation alone (Chuang et al., 2016). Therefore, from the perspective of theory of resource conservation, to meet individual innovation needs, employees must communicate and cooperate with other knowledge sources to enhance their own resources and capabilities (Huang, 2010). On the one hand, knowledge acquisition helps employees to absorb external available information resources, enrich the accumulation of original knowledge, and compensate for their own knowledge deficiency (Cui and Wu, 2016). Thus, the generation and practice of innovative ideas is promoted. On the other hand, knowledge acquisition can facilitate knowledge reorganization in employees, helping them acquire external knowledge and potential opportunities, thus stimulating the implementation of innovative behaviors. In addition, knowledge acquisition is conducive to reducing knowledge barriers and providing employees with more inspiration for innovation. Specifically, the expression and combination of diverse knowledge is conducive to the divergence of employees’ thinking and promotes the formation of new ideas (Crl et al., 2019). Based on the above analysis, this study suggests that challenge-hindrance stress affects innovation performance through knowledge acquisition. Therefore, the following hypotheses are proposed:

***Hypothesis 3a:*** *The positive association of challenge stress on innovation performance is mediated by knowledge acquisition.*

***Hypothesis 3b:*** *The negative association of hindrance stress on innovation performance is mediated by knowledge acquisition.*

### Chain Mediating Role of Knowledge Acquisition and Intrinsic Motivation

Based on transactional theory of stress and theory of resource conservation, the above analysis indicates that challenge stress enhances employees’ intrinsic motivation and promotes their knowledge acquisition, thus positively affecting their innovation performance. As a negative job stress, hindrance stress will reduce employees’ intrinsic motivation and enthusiasm for knowledge acquisition, thereby inhibiting the level of innovation performance. Based on this, the following hypotheses are proposed:

***H4a:*** *Intrinsic motivation and knowledge acquisition act as chain mediating factors in the positive relationship between challenge stress and innovation performance.*

***H4b:*** *Intrinsic motivation and knowledge acquisition act as chain mediating factors in the negative relationship between hindrance stress and innovation performance.*

## Materials and Methods

### Sample and Procedure

A simple random sampling method was used to select R&D technicians from six high-tech enterprises in Hunan Province as survey respondents. Based on the roster of R&D technicians personnel provided by the HR department, the questionnaire was distributed with the assistance of the HR department using the principle of voluntary participation. To reduce common method bias, this study adopted a three-point survey method (T1, T2, and T3) lasting for 3 months. A total of 380 questionnaires were distributed for each survey and there was a 1-month interval between each data collection. Prior to the official survey, the research team conducted a survey presentation to explain the details of the research. The questionnaire was anonymous and the data obtained were only used for scientific research. All data information will remain strictly confidential. The details are as follows: First, the researchers explained the precautions for completing the questionnaire to the respondents. Then, they filled out the paper questionnaire and only those who had completed the previous survey were allowed to proceed to the next survey. Finally, completed questionnaires were placed in a sealed bag by the researcher. The survey was conducted among 380 employees, and after removing questionnaires that were incomplete (because the participants withdrew in the middle, provided inconsistent answers, or obvious trending answers), a total of 295 valid sample data were collected, resulting in an effective rate of 77.63%. In stage T1, data on challenge stress, hindrance stress, and demographic variables were mainly collected. In stage T2, data on intrinsic motivation and knowledge acquisition were collected. In stage T3, data on innovation performance were collected. The specific demographic information shows that 64.41% of respondents are male, 54.24% younger than 30 years, and 88.13% hold a bachelor’s degree or above. More detailed information can be found in Table 1.

**TABLE 1:** Sample demographics (*N* = 295).

Characteristics	Demographic	Frequency	Percent
Gender	Male	190	64.41%
	Female	105	35.59%
Age(year)	≤29	160	54.24%
	30–39	88	29.83%
	40–49	38	12.88%
	≥50	9	3.05%
Education	Middle school or below	2	0.68%
	High school or secondary school	7	2.37%
	Junior college	26	8.82%
	Bachelor’s degree	192	65.08%
	Master’s degree or above	68	23.05%

### Measure

This study followed the translation-back translation procedure proposed by Brislin (1986). We translated the English scales into Chinese and then back-translated them to ensure the accuracy of the statements. All variables were measured in this study by anonymous self-assessment and Likert 5-point scale, ranging from 1 = strongly disagree to 5 = strongly agree.

#### Challenge Stress

Challenge stress were measured with a six-items scales (Cavanaugh et al., 2000). A sample item is “It takes me a lot of time to finish my work/tasks.” The scale’s alpha reliability in this study is 0.748.

#### Hindrance Stress

Hindrance stress were measured with a five-items scales (Cavanaugh et al., 2000). A sample item is “My work involves red tape and complicated procedures.” The scale’s alpha reliability in this study is 0.904.

#### Intrinsic Motivation

Intrinsic motivation were measured with a three-items scales (Blais et al., 1993). A sample item is “I’m really enjoying my job.” The scale’s alpha reliability in this study is 0.791.

#### Knowledge Acquisition

Knowledge acquisition were measured with a four-items scales (Chuang et al., 2016). A sample item is “I pay close attention to developments in my professional field.” The scale’s alpha reliability in this study is 0.898.

#### Innovation Performance

Innovation performance were measured with a nine-items scales (Janssen and Yperen, 2004). A sample item is “In my line of work, I’m creative and everyone learns from me.” The scale’s alpha reliability in this study is 0.919.

#### Control Variables

Following previous research (Webster et al., 2010; Granta and Berry, 2011), the demographic control variables measured were gender (coded 1 = male; 2 = female), age (coded 1 = 29 or below; 2 = between 30 and 39; 3 = between 40 and 49; 4 = 50 and above), education experience (coded 1 = middle school or below; 2 = high school or secondary school; 3 = junior college; 4 = Bachelor’s degree; 5 = Master’s degree or above).

### Data Analysis

The data analysis process for this study was as follows. First, validation factor analysis was performed using Amos 22.0 to test the discriminant validity between challenge stress, hindrance stress, intrinsic motivation, knowledge acquisition, and innovation performance. Second, Descriptive statistical analysis and correlation analysis were performed using SPSS 22.0. Third, the SPSS PROCESS Macro (Hayes, 2013) was used to test the hypotheses.

## Results

### Exploratory Factor Analysis

Table 2 shows that the alpha reliability values of all variables of the scale exceed 0.7, indicating that the scale has good reliability. Moreover, to verify the validity of the scale, the data were analyzed for convergent and discriminant validity. As shown in Table 2, the standardized factor loading of all variables exceeded 0.6, and both composite reliability (CR) and average variance extracted (AVE) exceeded their acceptable values of 0.7 and 0.5, respectively, indicating that the scale has good discriminative validity and convergent validity.

**TABLE 2:** Results for reliability and validity.

Variables	Item	Factor loadings	α	AVE	CR
CS	CS1	0.719	0.748	0.569	0.887
	CS2	0.783			
	CS3	0.706			
	CS4	0.829			
	CS5	0.769			
	CS6	0.714			
HS	HS1	0.771	0.904	0.584	0.875
	HS2	0.755			
	HS3	0.820			
	HS4	0.733			
	HS5	0.740			
IM	IM1	0.688	0.791	0.533	0.773
	IM2	0.789			
	IM3	0.711			
KA	KA1	0.735	0.898	0.573	0.843
	KA2	0.733			
	KA3	0.749			
	KA4	0.810			
IP	IP1	0.816	0.919	0.567	0.921
	IP2	0.809			
	IP3	0.728			
	IP4	0.762			
	IP5	0.749			
	IP6	0.721			
	IP7	0.718			
	IP8	0.704			
	IP9	0.762			

*CS, challenge stress; HS, hindrance stress; IM, intrinsic motivation; KA, knowledge acquisition; IP, innovation performance.*

### Confirmatory Factor Analysis

According to Podsakoff et al. (2003), the Harman single factor method was used to test for common variance. After factor analysis with SPSS22.0 statistical analysis software, the total variance explained measure was 61.30%. Five factors with characteristic roots greater than 1 were obtained, and the variance of the explanation of the first factor was 16.51%, which is lower than 30%. There is no serious common method bias problem. Moreover, confirmatory factor analysis (CFA) was used to test the discriminant validity of key variables. The study examined a baseline model that contains five factors; namely, challenge stress, hindrance stress, intrinsic motivation, knowledge acquisition, and innovation performance. Table 3 shows the results of CFA. Compared with other models, all indexes of the five-factor model had the best model fit: *X*^2^/*df* = 2.057, RMSEA = 0.058, SRMR = 0.061, IFI = 0.935, TLI = 0.931, and CFI = 0.937. Therefore, the five-factor model has good discriminative validity and outperformed other models.

**TABLE 3:** Confirmatory factor analysis results.

Factor model	X^2^/df	RMSEA	SRMR	IFI	TLI	CFI
5 factor model (CS, HS, IM, KA, IP)	2.057	0.058	0.061	0.935	0.931	0.937
4 factor model (CS, HS, IM + KA, IP)	2.861	0.069	0.074	0.882	0.810	0.793
3 factor model (CS, HS, IM + KA + IP)	3.617	0.072	0.086	0.710	0.699	0.722
2 factor model (CS + HS, IM + KA + IP)	4.220	0.083	0.102	0.694	0.621	0.648
1 factor model (CS + HS + IM + KA + IP)	4.935	0.091	0.141	0.602	0.517	0.596

*N = 295. CS, challenge stress; HS, hindrance stress; IM, intrinsic motivation; KA, knowledge acquisition; IP, innovation performance.*

### Descriptive Statistical Analysis

Table 4 presents the means, standard deviations and correlations of all the variables. Challenge stress is positively correlated with innovation performance (γ = 0.369, *p* < 0.001), and intrinsic motivation (γ = 0.473, *p* < 0.001), and knowledge acquisition (γ = 0.322, *p* < 0.001). Hindrance stress is negatively correlated with innovation performance (γ = –0.393, *p* < 0.001), and intrinsic motivation (γ = –0.266, *p* < 0.001), and knowledge acquisition (γ = –0.253, *p* < 0.001). The above data preliminarily support the hypothesis of main effect and mediating effect in this study.

**TABLE 4:** Means, standard deviation, and correlations among variables.

Variable	1	2	3	4	5	6	7	8
(1) Sex	1							
(2) Age	–0.129*	1						
(3) Education	0.015	0.026	1					
(4) CS	0.045	–0.047	0.017	1				
(5) HS	–0.048	0.063	0.019	–0.405***	1			
(6) IM	0.075	0.076	0.078	0.473***	–0.266***	1		
(7) KA	0.071	–0.059	0.112	0.322***	–0.253***	0.313***	1	
(8) IP	0.034	–0.148*	0.037	0.369***	–0.393***	0.340***	0.273***	1
Average mean	1.513	1.608	2.831	2.727	2.460	2.273	2.773	3.192
Standard deviation	0.501	0.898	0.613	0.678	0.901	0.754	0.941	0.944

N = 295. *p < 0.05, ***p < 0.001. CS, challenge stress; HS, hindrance stress; IM, intrinsic motivation; KA, knowledge acquisition; IP, innovation performance.

### Hypothesis Testing

Based on the Process procedure recommended by Preacher and Hayes (2011) and the Bootstrap method, the chain mediating variable hypothesis test was performed to improve the robustness. Specifically, this study selected model 4 and Model 6 in the Process program, the Bootstrap sampling number was set at 5000, the confidence level of the confidence interval was set at 95%. And the independent variables, mediating variables, outcome variables and control variables were put into the Process procedure simultaneously. All the results required are obtained at one time, including the results of the path coefficients between the variables and the results of the indirect effects. If the 95% confidence interval for these path coefficients does not contain 0, this indicates a significant mediating effect.

**FIGURE 2:**
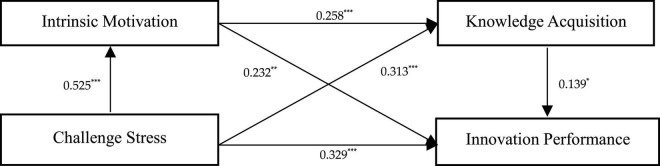
Main effect and indirect effect. *n* = 376; *p < 0.05; **p < 0.01; ***p < 0.001.

**FIGURE 3:**
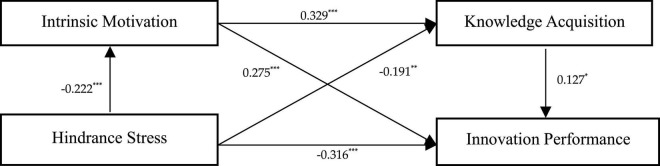
Main effect and indirect effect. *n* = 376; *p < 0.05; **p < 0.01; ***p < 0.001.

The direct association of challenge-hindrance stress on innovation performance was tested. As shown in Table 5 and Figure 2, a significant positive association of challenge stress on innovation performance was found (*b* = 0.329, CI = [0.161, 0.496]). The confidence interval does not contain 0, which indicates that challenge stress the positive association with innovation performance is significant, thus supporting Hypothesis 1a. As shown in Table 5 and Figure 3, a significant positive association of hindrance stress with innovation performance was found (*b* = –0.316, CI = [–0.429, –0.204]). The confidence interval does not contain 0, which indicates that hindrance stress has a negative association with innovation performance, thus supporting Hypothesis 1b.

**TABLE 5:** Hypothesis test results.

CS→IM→KA→IP				HS→IM→KA→IP			

**Path**	***b***	**Bootstrap 95% CI**		**Path**	***b***	**Bootstrap 95% CI**	
		**LLCI**	**UPCI**			**LLCI**	**UPCI**
Total effect	0.513	0.364	0.661	Total effect	–0.412	–0.523	–0.301
Direct effect (CS→IP)	0.329	0.161	0.496	Direct effect (HS→IP)	–0.316	–0.429	–0.204
Total indirect effect	0.184	0.086	0.284	Total indirect effect	–0.095	–0.147	–0.050
Indirect effect				Indirect effect			
CS→IM→IP	0.122	0.035	0.207	HS→IM→IP	–0.062	–0.103	–0.027
CS→KA→IP	0.043	0.007	0.090	HS→KA→IP	–0.024	–0.053	–0.003
CS→IM→KA→IP	0.018	0.002	0.040	HS→IM→KA→IP	–0.009	–0.020	–0.001

*CS, hindrance stress; HS, hindrance stress; IM, intrinsic motivation; KA, knowledge acquisition; IP, innovation performance.*

Hypotheses 2a and 2b predict that intrinsic motivation mediates the relationship between challenge-hindrance stress and innovation performance. The mediating role of intrinsic motivation was examined using the process procedure and Model4 in the Bootstrap method. As shown in Figure 2, challenge stress was positively correlated with intrinsic motivation (β = 0.525, *p* < 0.001), and intrinsic motivation was positively correlated with innovation performance (β = 0.232, *p* < 0.01). As shown in Table 5, Bootstrap analysis showed that intrinsic motivation has a mediating role in the relationship between challenge stress and innovation performance, and the confidence interval does not contain 0 (b = 0.122, CI = [0.035, 0.207]), thus supporting Hypothesis 2a. As shown in Figure 2, hindrance stress was negatively correlated with intrinsic motivation (β = –0.222, *p* < 0.001), while intrinsic motivation was positively correlated with innovation performance (β = 0.275, *p* < 0.001). As shown in Table 5, intrinsic motivation has a mediating role in the relationship between hindrance stress and innovation performance, and the confidence interval does not contain 0 (*b* = –0.062, CI = [–0.103, –0.027]), thus supporting Hypothesis 2b.

Hypotheses 3a and 3b predict that knowledge acquisition mediates the relationship between challenge-hindrance stress and innovation performance. Similarly, the mediating role of knowledge acquisition was assessed using the Process procedure and Model4 in the Bootstrap method. As shown in Figure 2, challenge stress was positively correlated with knowledge acquisition (β = 0.313, *p* < 0.001), and knowledge acquisition was positively correlated with innovation performance (β = 0.139, *p* < 0.05). As shown in Table 5, knowledge acquisition has a mediating role in the relationship between challenge stress and innovation performance, and the confidence interval does not contain 0 (*b* = 0.043, CI = [0.007, 0.090]), thus supporting Hypothesis 3a. Moreover, as shown in Table 5, knowledge acquisition has a mediating role in the relationship between hindrance stress and innovation performance, and the confidence interval does not contain 0 (*b* = –0.024, CI = [–0.053, –0.003]), thus supporting Hypothesis 3b.

Finally, the chain mediating association of the knowledge acquisition and intrinsic motivation in the relationship between challenge-hindrance stress and innovation performance was examined. The chain mediating roles of intrinsic motivation and knowledge acquisition were examined using the Process procedure and Model6 in the Bootstrap method. As shown in Table 5, the chain mediating association of intrinsic motivation and knowledge acquisition on the relationship between challenge stress and innovation performance was significant, and the confidence interval does not contain 0 (*b* = 0.018, CI = [0.002, 0.040]), thus supporting Hypothesis 4a. In the same way, the chain mediating association of intrinsic motivation and knowledge acquisition on the relationship between hindrance stress and innovation performance was significant, and confidence interval does not contain 0 (*b* = –0.009, CI = [–0.020, –0.001]), thus supporting Hypothesis 4b.

## Discussion

In this study, 295 sample data were collected at three time points. Based on the transactional theory of stress and theory of resource conservation, the impact mechanism of challenge-hindrance stress on employees’ innovation performance is explored. On the one hand, the different impact of challenge-hindrance stress on innovation performance is shown. On the other hand, intrinsic motivation and knowledge acquisition are found to play a mediating role in the model. The research results not only expand the research perspective on job stress, but also analyze the internal mechanism between job stress and innovation performance.

### Theoretical Contribution

Several theoretical implications can be gained from this study. First, the double-edged effect of job stress on innovation performance has been demonstrated, whereby challenge stress has a positive association with innovation performance and hindrance stress has a negative effect with innovation performance. Previous studies mainly analyzed the outcome effect of job stress from a single perspective (Wallace et al., 2009; Montani et al., 2017), whereby it remained unclear whether job stress introduces a double-edged sword effect. From the perspective of the nature of job stress, this paper classifies job stress into challenge stress and hindrance stress and explores their different impacts on innovation performance. The findings expose the reasons for the double-edged sword effect of job stress and overcome the limitations of previous studies.

The second theoretical contribution confirms the mediating role of intrinsic motivation between challenge-hindrance stress and innovation performance. Previous research explored the relationship between challenge-hindrance stress and innovation performance as well as the impact of intrinsic motivation on innovation performance (Min et al., 2015; Yun et al., 2019). However, few studies have integrated these constructs under the same theoretical framework. While the mechanism of how job stress affects innovation performance has been explained from the perspectives of leadership style, organizational environment, and organizational support (Micevski et al., 2019; Tan et al., 2020), little attention has been paid to the role of intrinsic motivation in mediating between the two. Based on the theory of resource conservation, this study uncovers the mediating role of intrinsic motivation in the relationship between challenge-hindrance stress and innovation performance. Therefore, this study extends the research perspective of mediating mechanisms in the relationship between job stress and innovation performance.

The third theoretical contribution confirms the mediating role of knowledge acquisition in the relationship between challenge-hindrance stress and innovation performance. Previous studies mainly analyzed antecedent variables of knowledge acquisition (Wang and Zhang, 2010; Martinez et al., 2012), while the mediating role of knowledge acquisition, particularly the mediating role of knowledge acquisition in the relationship between challenge-hindrance stress and innovation performance has not been considered. The present research identified the knowledge acquisition process mechanism between job stress and innovation performance, thus enriching the research achievements in the field of knowledge acquisition.

The fourth theoretical contribution is the proof that intrinsic motivation and knowledge acquisition play a chain mediating role in the process of challenge- hindrance stress affecting innovation performance. Based on previous research results, intrinsic motivation and knowledge acquisition are introduced as mediating variables. The results indicate that job stress affects innovation performance through both intrinsic motivation and knowledge acquisition. This finding enriches the theoretical understanding of the logical relationship between the antecedent variables of innovation performance, and thus helps subsequent studies to better select proximal antecedents of innovation performance. Moreover, this study also provides useful insights for future research on the mediating role of the test chain.

### Practical Implications

First, this study confirms that challenge stress promotes innovation performance, while hindrance stress inhibits innovation performance. These findings lead to the recommendation that managers can use challenge stress in the tasking process (as appropriate) to stimulate employee creativity. It is important to focus on the management of hindrance stress. On the one hand, organizations should eliminate the generation of hindrance stress from the source and create a good working atmosphere for their employees. On the other hand, the organization should focus on managing the daily job stress of employees, helping them to resolve difficulties, and eliminating negative emotions. Moreover, while managers focus on stress levels, they should also distinguish between “good” and “bad” stress, rather than blindly dismissing the effects of stress.

Second, this study confirms that intrinsic motivation mediates the relationship between challenge-hindrance stress and innovation performance, which prompts managers to pay close attention to employees’ psychological and emotion states in time, and emphasize the guidance of intrinsic motivation. From a recruitment and training perspective, companies can use personality tests and stress interviews to assess a candidate’s stress tolerance, thus providing a reference for selection and promotion of executives. Furthermore, through ideological and skills training employees should be guided to regard job stress positively.

Finally, this study confirms that knowledge acquisition mediates the relationship between challenge-hindrance stress and innovation performance. This suggests that managers should broaden the channels of knowledge acquisition in organizations and stimulate employees’ intrinsic motivation for knowledge acquisition (Chuang et al., 2016). On the one hand, companies should build an information acquisition platform to broaden the channels of knowledge sources, provide real-time information and resources for employees, and increase their knowledge reserves. On the other hand, companies should focus on the management of employees’ knowledge acquisition, incorporate knowledge acquisition into the organizational performance appraisal system to enhance employees’ motivation to acquire external resources, and thus improve innovation performance.

### Limitations and Future Research

This study has the following three limitations: The first is a limitation of the research design. This study only samples R&D personnel, resulting in a lack of generalizability of the findings. Future research can consider investigating other industry personnel to enhance the generalizability of findings. Moreover, the research data originates from employees’ self-reports. Although three time periods and anonymous reporting were used to collect data, common method bias cannot be completely avoided. Future research can collect data through multi-source questionnaire, experimental methods, or interview method to avoid common method bias.

The second limitation is one of variable selection. Although this study confirms the mediating association of intrinsic motivation and knowledge acquisition between challenge-hindrance stress and innovation performance, only the mediating mechanism is explored without analyzing its boundary conditions. Future research can incorporate both mediating variables and moderating variables into the theoretical framework of challenge-hindrance stress. For example, the moderating effects of organizational climate or leadership support in the relationship between challenge-hindrance stress and innovation performance can be explored.

Third, the limitations of theoretical options. This research develops a theoretical model analysis based on the transactional theory of stress and theory of resource conservation. Future research could analyze the mechanisms of action between job stress and innovation performance from other theoretical perspectives, such as emotional event theory, social learning theory, and balance theory, thus enrich the explanatory mechanisms of the outcome effects of job stress.

## Data Availability Statement

The original contributions presented in the study are included in the article/supplementary material, further inquiries can be directed to the corresponding author/s.

## Ethics Statement

The studies involving human participants were reviewed and approved by Ethics Committee of Experimental Animals, South China University of Technology. The patients/participants provided their written informed consent to participate in this study.

## Author Contributions

SY and CX conceived and designed the work. CX and XG collected the data. XG, WC, and SY analyzed and interpreted the data. XG and CX drafted the manuscript. WC and CX were responsible for the modifications and checked the final version of the manuscript. All authors contributed to the article and approved the submitted version.

## Conflict of Interest

The authors declare that the research was conducted in the absence of any commercial or financial relationships that could be construed as a potential conflict of interest.

## Publisher’s Note

All claims expressed in this article are solely those of the authors and do not necessarily represent those of their affiliated organizations, or those of the publisher, the editors and the reviewers. Any product that may be evaluated in this article, or claim that may be made by its manufacturer, is not guaranteed or endorsed by the publisher.
